# Impact of the new European medical device regulation on digital health manufacturers

**DOI:** 10.1038/s41746-026-02812-1

**Published:** 2026-05-26

**Authors:** Liga Svempe

**Affiliations:** Riga Stradiņš University, Faculty of Social Sciences, Riga, Latvia

**Keywords:** Technology, Industry, Business, Law

## Abstract

This study assessed the Medical Device Regulation EU 2017/745 impact on the digital health companies and the industry. An online survey was conducted in April-May 2025, targeting manufacturers registered in the EUDAMED database with a software-type product. 35 responses were received. The survey results empirically confirm the negative effect of the theoretically identified hindering factors. The financial burden was the most common challenge, with 31 respondents (88.6%) reporting a significant or severe impact. The regulation causes delays, the new clinical evidence requirements are challenging, and the process requires more resources. 6 companies (23.1% of those planning a launch) have canceled a new product launch, while 8 (34.8% of companies with 2 or more products) have discontinued an existing product. 6 respondents (17.1%) are exiting the EU market, and 10 companies (28.6%) are considering shutting down their operations. 16 respondents (45.7%) consider certification not worthwhile if the buyers are individual consumers.

## Introduction

Rapid technological advancements have transformed the healthcare industry over the past decades. Today, numerous digital solutions enter the market, aiming to diagnose, monitor, or treat medical conditions and ultimately improve patients’ quality of life. However, there were concerns regarding whether the previous EU regulations (the Medical Device Directive 93/42/EEC^1^, hereinafter - MDD) were sufficient to ensure patient safety, especially due to various scandals that exposed gaps in regulatory oversight. The most notable recall was the Poly Implant Prothese scandal, where the manufacturer used non-medical grade silicone, putting at risk thousands of recipients worldwide^2^. In response, the European Union revised its medical device regulations in 2017 and introduced the Medical Device Regulation (EU) 2017/745^3^ (hereinafter - MDR) to strengthen oversight and enhance patient protection. Here the definition of a medical device included software as a form of device and introduced a specific risk classification rule for such devices (Rule 11). With the advancements of AI, an increasing number of Software as a Medical Device (SaMD) products incorporate AI technologies, bringing also additional risks^4^, so all of these devices have to be compliant with the current regulations.

The new MDR introduced stricter requirements for medical devices, covering development, quality assurance, clinical evaluation, and post-market surveillance. However, the revised framework also sparked discussions about its actual impact on innovation, with concerns that it may slow technological advancement and reduce the availability of medical devices, potentially undermining its initial goal of improving patient safety. This is especially important, as the AI advancements in the past years have been tremendous, holding a transformative power^5,6^. The stringent requirements can be particularly challenging for SMEs, which often lack the financial resources to navigate the complex regulatory landscape, making market entry more difficult. For companies with existing products, the new regulation may result in product upclassification, which means that the product’s risk class is changed to a higher class. Consequently, the company must comply with more stringent regulatory requirements, which can require more resources. For some companies, the regulatory burden can result in leaving the EU market or shutting down business operations. According to 2024 data from MedTech Europe^7^, SMEs account for 90% of medtech companies, highlighting the widespread impact of these regulatory challenges.

Previous research suggests that the new regulation poses considerable challenges for SaMD manufacturers, particularly for SMEs. However, the scope of existing studies remains limited. A pilot study^8^ with a convenience sample explored the impact; however, it also acknowledged the limitations in the generalizability of its findings. Additionally, that survey took place in 2017, before the full adoption of the MDR, making the results less comprehensive, yet it serves as an initial basis for exploring the MDR’s impact. Another study, published in 2023, examined the perspectives of German orthopedic aid manufacturers^9^, however, it is limited to a specific industry and one country. While many of its findings may be relevant across industries and geographies, the impact on the digital health industry and its specifics remains largely unexplored.

The author previously conducted a theoretical study identifying hindering factors, which were grouped into eight dimensions/domains^10^: (1) more complex requirements leading to delays, (2) more requirements for clinical trials, (3) an increase in expenses, (4) classification issues, (5) limited availability of Notified Bodies (hereinafter - NB), (6) lack of knowledge, (7) lack of guidance, and (8) constraints on software updates. This paper explores the practical impact of the new MDR from the manufacturers’ perspective and aims to assess whether and to what extent these hindering factors impact product development, thereby creating a comprehensive perspective on this matter. It also helps to determine which domains should be prioritized for actionable initiatives to mitigate their negative effects and may serve as guidance for policymakers. The theoretically identified hindering factors and outcomes from the previous study served as the foundation for formulating the survey questions for this study.

Additionally, the author hypothesizes that for many manufacturers, especially those developing low-risk tools, it is a strategic business decision to avoid the certification pathway and work in the wellness industry due to the regulatory complexity if the buyer is an individual consumer. However, this approach can lead to a situation where the market is overflooded with low-quality tools that can even be harmful for the end user^11^. To provide evidence that the type of buyer persona influences the decision to pursue certification, the survey includes one question to explore this matter.

The author defines three Research questions:

1. What is the impact of the new MDR on digital health manufacturers’ business operations?
2. What are the consequences of the MDR on digital medical device availability?
3. Does the type of buyer persona influence the decision to pursue medical device certification?

## Results

In April 2025, the first email was sent out to 691 recipients. Out of those, 37 emails were unreachable due to various reasons - the email was not found, the domain did not exist, and others. In total, 35 responses were received (response rate 5.35%).

The characteristics of the respondents are summarized in Table 1. The most responses were received from Finland and France, and the most popular markets were Germany, the UK, and France, followed by Italy and the USA. The proportion of SMEs was consistent with the industry structure, with 88.6% classified as SMEs - closely aligning with general data indicating that 90% of medical technology companies are SMEs^7^.

**Table 1:** Characteristics of respondents (*n* = 35)

*Characteristics*	*Value, n (%)*
Country of origin:	
Finland	6 (17.1%)
France	5 (14.3%)
United Kingdom	4 (11.4%)
Austria, Germany, Netherlands	3 (8.6%) each
Belgium, Italy, Poland, Spain	2 (5.7%) each
Czech Republic, Latvia	1 (2.9%) each
Other	1 (2.9%)
Main markets:	
Germany	20 (57.1%)
United Kingdom	19 (54.3%)
France	18 (51.4%)
Italy, USA	15 (42.9%)
Poland, Spain, Switzerland	10 (28.6%) each
Belgium	9 (25.7%)
Netherlands	8 (22.9%)
Austria, Portugal	7 (20%)
Finland, Ireland, Sweden	6 (17.1%) each
Denmark	5 (14.3%)
Czech Republic, Greece	4 (11.4%) each
Luxembourg	3 (8.6%)
Croatia, Estonia, Iceland, Latvia, Malta, Norway, Slovenia	2 (5.7%) each
Bulgaria, Hungary, Liechtenstein, Lithuania, Romania,	1 (2.9%) each
Slovakia	0 (0%)
Cyprus	7 (20%)
Other	
Company size:	
SME	31 (88.6%)
Large	4 (11.4%)

The following sub-chapters examine the research findings in detail to assess whether they support the theoretically identified hindering factors.

### Hindering factors

**Fig. 1:**
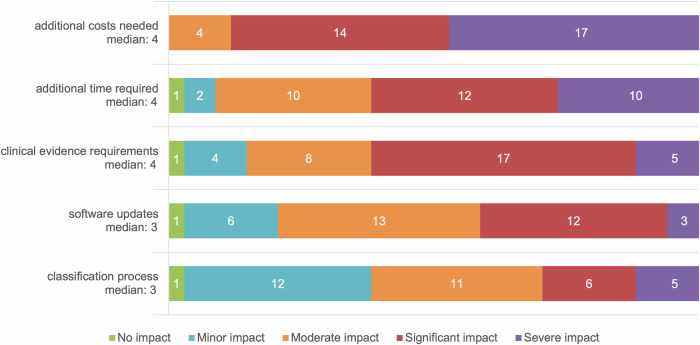
Participants’ agreement with statements regarding the impact of the regulation. Median is calculated by providing a numeric value to the responses (1 = no impact; 5 = severe impact).

The three most significant factors reported were increased costs, additional time, and clinical evidence requirements, each with a median impact rating of 4 (“Significant impact”). Among these, the financial burden was the most widely experienced challenge: 31 (88.6%) of respondents indicated that the additional costs had a significant or severe impact on their business operations (Fig. 1). This is followed by additional time needed and more requirements for clinical evidence, reported as having a significant or severe impact by 22 (62.9%) respondents. Among the five hindering factors, classification process issues were the least impactful, with 11 respondents (31.4%) reporting a significant or severe impact. Nevertheless, all the factors demonstrated a noteworthy overall impact, as only one respondent in four of the five domains reported experiencing no impact.

Stacked bar chart comparing how different regulatory factors impact manufacturers. Green represents “no impact”, blue “minor impact”, orange “moderate impact”, red “significant impact”, and purple “severe impact”. Each bar shows how many respondents selected each impact level, with the median impact score displayed to the left. The median is calculated by assigning numerical values to responses (1 = no impact; 5 = severe impact).

Compared to the 2017 survey^8^, the two main challenges - additional costs and the additional time required - remain unchanged. This indicates that even several years after the adoption of the MDR, companies continue to face the same issues, and stakeholders should therefore prioritize addressing these factors.

**Fig. 2:**
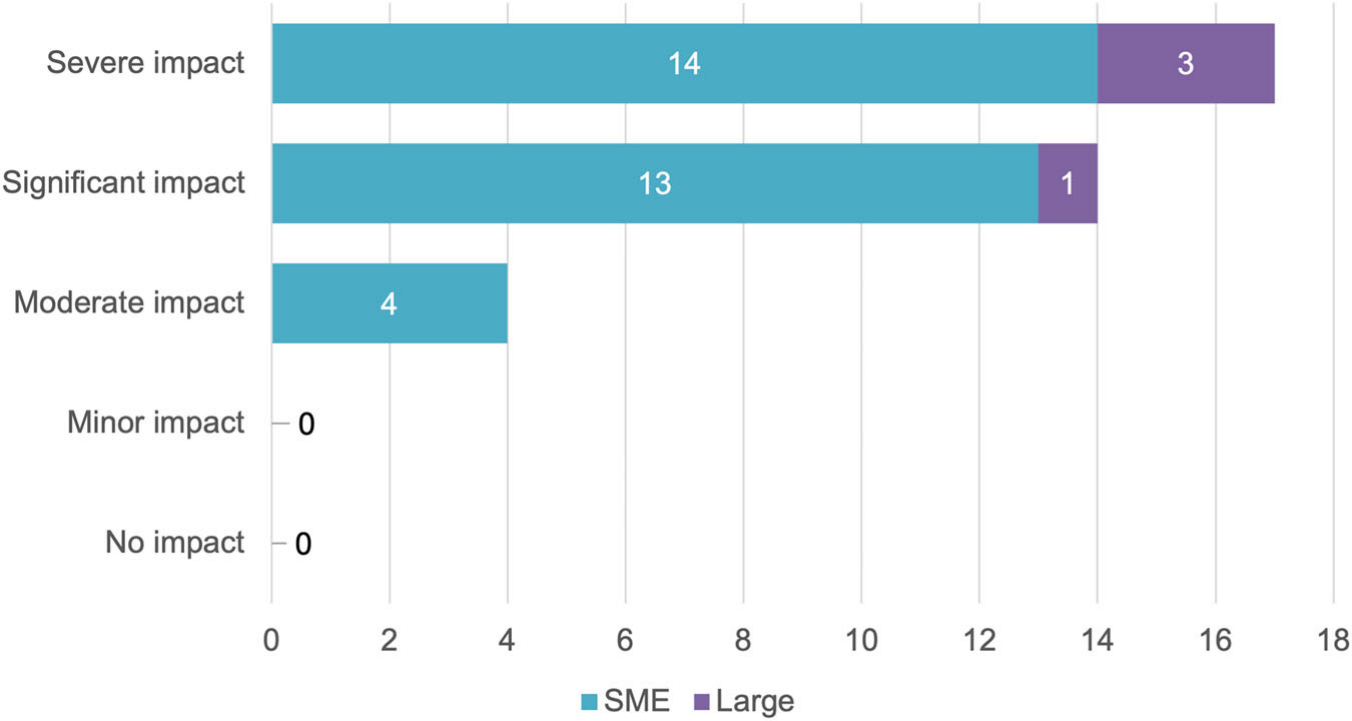
The impact of the MDR on adding costs. Number of respondents experiencing the respective level of impact.

The burden of additional costs is not limited to SMEs. Large companies have also reported being significantly or severely affected (see Fig. 2). No respondent reported having no or a minor impact.

Bar chart illustrating how the MDR impacts manufacturers by adding more costs. The chart displays the distribution of respondents across impact levels - from no impact to severe impact. Blue represents SMEs, purple represents large companies. Each bar shows how many respondents selected each impact level.

**Fig. 3:**
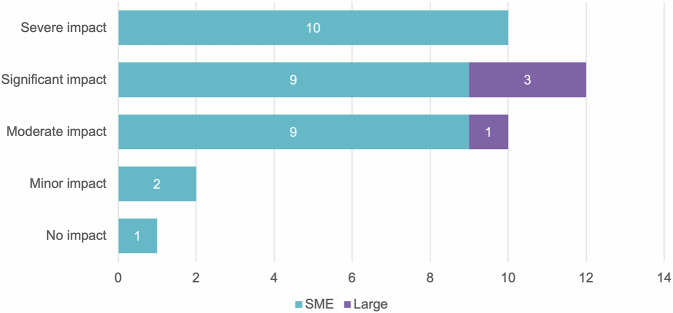
The impact of the MDR on additional time needed to launch a SaMD. Number of respondents experiencing the respective level of impact.

The second most significant factor was the additional time required to bring a product to market. Notably, large companies are not immune to this challenge as well (Fig. 3). While it might be assumed that such companies possess greater resources (financial, talent, and other) to launch the products quicker, they also reported the extended timelines as having a significant or moderate impact.

Bar chart illustrating how the impact of the MDR on manufacturers, requiring more time to launch medical devices. The chart displays the distribution of respondents across impact levels - from no impact to severe impact. Blue represents SMEs, purple represents large companies. Each bar shows how many respondents selected each impact level.

**Fig. 4:**
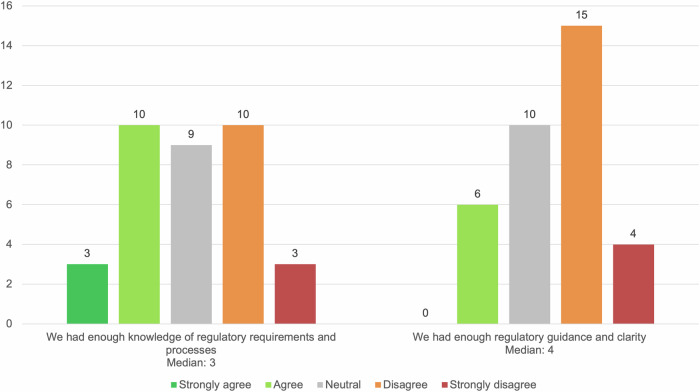
Participants’ agreement with statements regarding the regulatory knowledge and guidance. Median is calculated by providing a numeric value to the responses (1 = Strongly agree; 5 =Strongly disagree).

Lack of knowledge and insufficient regulatory guidance were another two hindering factors. Survey results indicate that an equal number of companies (13 or 37.1%) reported either having sufficient knowledge of regulatory requirements and processes or having a lack of it (Mdn=3; Fig. 4).

Bar chart displaying the agreement level with the statements regarding the regulatory knowledge and guidance if it was sufficient. Green represents “strongly agree”, light green “agree”, gray “neutral”, orange “disagree”, and red “strongly disagree”. Each bar shows how many respondents selected each level, with the median impact score of each factor displayed below. The median is calculated by assigning numerical values to responses (1 = strongly agree; 5 = strongly disagree).

However, less than half of that number of respondents indicated having enough regulatory guidance, with 19 respondents (54.3%) reporting a lack of it (Mdn=4, which equals “Disagree”). Notably, previous experience with the Medical Devices Directive (MDD) did not appear to mitigate these challenges - both companies familiar with the MDD and working only with the MDR reported gaps in knowledge (U = 108.000, Z = -1.235, *p* = 0.243) and guidance (U = 129.500, Z = -0.488, *p* = 0.649).

**Fig. 5:**
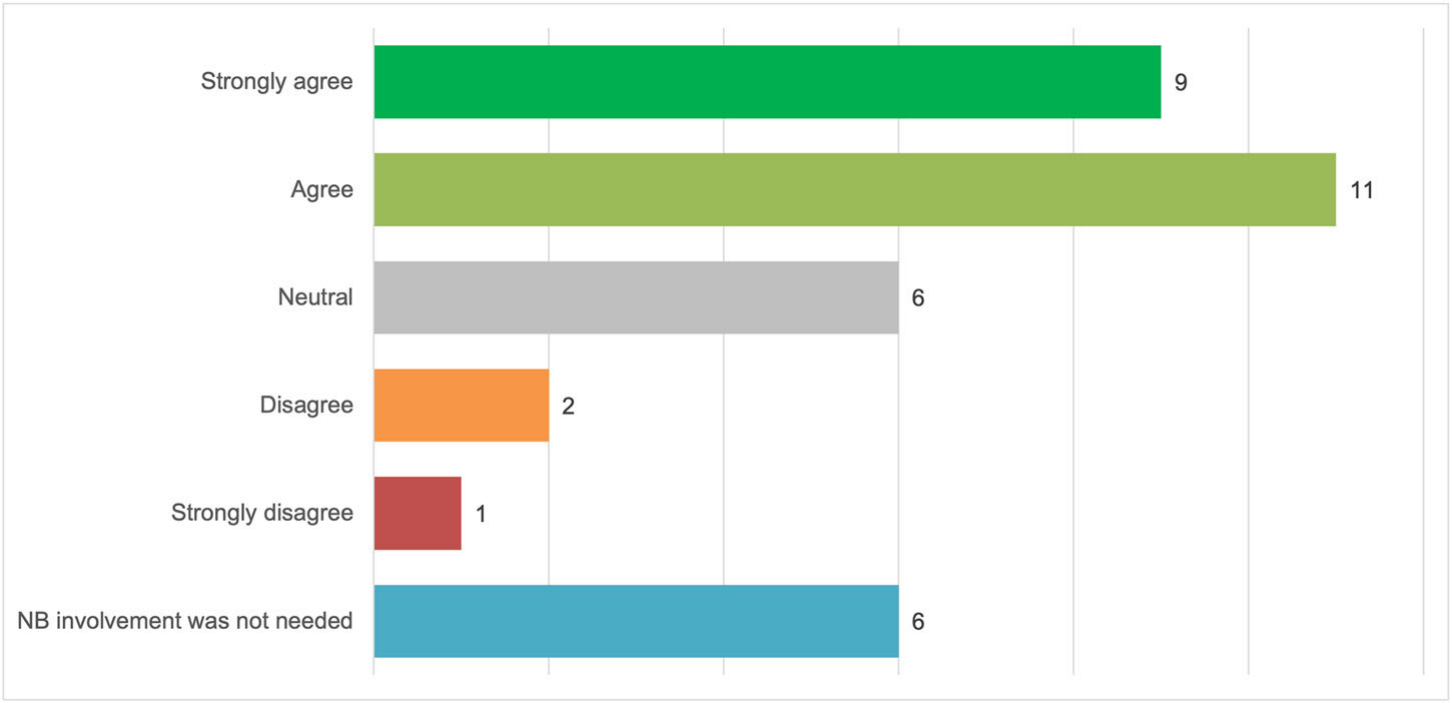
Participants’ agreement on experiencing problems with the Notified Bodies (Mdn = 2). Median is calculated by providing a numeric value to the responses (1 = Strongly agree; 5 =Strongly disagree; responses “NB involvement was not needed” were excluded).

The availability of Notified Bodies had been a challenge since the adoption of the regulation^12^, and it still remains a considerable hindering factor: 20 companies, representing 69% of those that required NB involvement, reported experiencing related challenges (Fig. 5; Mdn=2, which equals “Agree”).

Bar chart illustrating the level of agreement with a statement if the respondents experiences problems with the Notified Bodies. The chart displays the distribution of respondents across impact levels - from strongly agree to strongly disagree, and a separate bar in blue displays the number or respondents that did not require the NB involvement. Green represents “strongly agree”, light green “agree”, gray “neutral”, orange “disagree”, and red “strongly disagree”. Each bar shows how many respondents selected each agreement level. The median is calculated by assigning numerical values to responses (1 = Strongly agree; 5 =Strongly disagree; responses “NB involvement was not needed” were excluded).

**Fig. 6:**
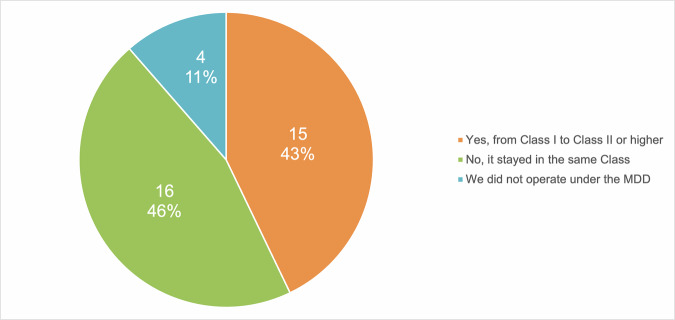
Participants’ responses whether their SaMD product was up-classified, compared to the MDD. Number of respondents and percentage of all respondents.

Another frequently debated concern is the ambiguity surrounding the classification process, and for many companies - the need to upclassify their products compared to the previous MDD framework^13^. The survey findings show that 48.4% of respondents who had operated under the MDD were required to upclassify their products from Class I to at least Class II (Fig. 6).

Chart illustrating how many companies had to up-classify their products due to the new regulation. The orange represents number of respondents who had to up-classify from Class I to Class II or higher, the green represents number of respondents whose devices remained in the same Class, and blue represents the number of companies that did not operate under the previous regulation (MDD), thus, did not have this experience. Each slice shows how many respondents selected each agreement level in numerical values and percentages.

This upclassification can be a daunting burden for developers, as it entails stricter requirements for clinical evaluation and reporting, and also requires assessment by a Notified Body, which can be challenging for many companies with limited resources. Analysis of the responses from companies whose products were upclassified from Class I to a higher class indicates that this change had a significant or severe impact across the three key domains: increased costs, additional time needed, and challenges in fulfilling clinical evidence requirements. No company reported that upclassification had no impact on any of the hindering factors. However, comparing this data to the responses from companies that did not have to upclassify, there is no statistically significant association between whether a device was upclassified and the reported impact levels: all the p-values are greater than 0.05 (for additional costs needed U = 99.000, Z = -0.919, *p* = 0.423; for additional time required U = 97.500, Z = -0.930, *p* = 0.379; and for clinical evidence requirements U = 93.500, Z = -1.136, *p* = 0.299). This means that additional costs, delays, and clinical evidence requirements were challenges for all companies, regardless of whether their product was upclassified or not.

### Consequences in the market

Numerous potential consequences of the new regulation on medical device availability and market dynamics have been theoretically identified and discussed^10^. The survey sought to empirically validate these claims.

Unfortunately, the findings affirm that the new regulation is causing companies to reduce their product portfolio and to shift their businesses in favor of other regions (Table 2). 6 companies (23.1% of those planning a launch) have already canceled a new product launch, while 8 companies have discontinued an existing product, which represents 34.8% of companies that have had two or more products at any point of their existance. Additionally, 6 companies (17.1%) are exiting the EU market in favor of other regions, and 10 companies (28.6%) are considering shutting down their operations.

**Table 2:** Participants’ responses on the consequences of the MDR on their business operations (*n* = 35)

	n	%
Have you canceled a new product launch due to the MDR?		
Yes	6	17.1%
No	20	57.1%
We had no plans to launch a new SAMD product	9	25.7%
Have you reduced your product portfolio (discontinuing at least one product) due to the MDR?		
Yes	8	22.9%
No	15	42.9%
We have always had only one product	12	34.3%
Have you exited or are you in the process of leaving the EU market for at least one product while continuing to sell that product in other markets?		
Yes	6	17.1%
No	29	82.9%
Are you planning, or at least considering, shutting down operations due to the regulatory burden?		
Yes	10	28.6%
No	25	71.4%

### Market Appeal of Certification

The author hypothesized that certification is less appealing to companies operating under a business-to-consumer (B2C) business model, as individual consumers tend to place little value or pay no attention to certification. This study aimed to validate that hypothesis empirically, and the findings support this claim (Research question no. 3).

**Fig. 7:**
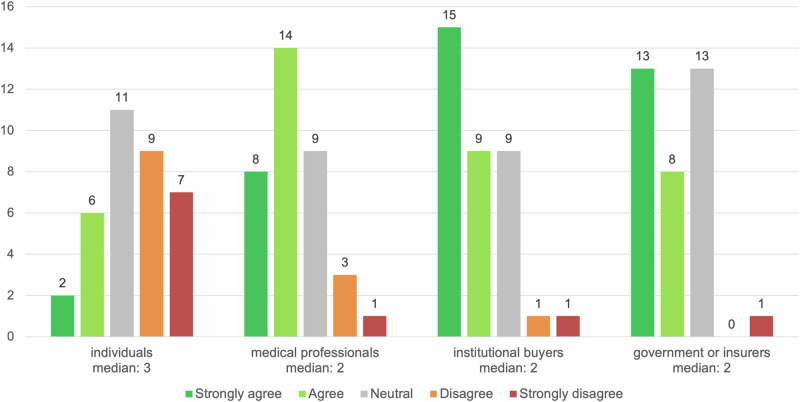
Participants’ agreement with statements on whether it is worthwhile to obtain CE certification, depending on the type of customer. Median is calculated by providing a numeric value to the responses (1 = strongly agree; 5 = strongly disagree).

As illustrated in Fig. 7, only 8 respondents (22.9%) agreed that obtaining CE certification is worthwhile when the end buyer is an individual consumer. At the same time, a significantly larger portion of respondents - 21 to 24 (60-68.6%) - agreed with this statement when the buyer is a medical professional, an institutional buyer, a government body, or an insurer.

Bar chart displaying the perspective of the respondents if obtaining the certification is worthwhile depending on the type of customer. Green represents “strongly agree”, light green “agree”, gray “neutral”, orange “disagree”, and red “strongly disagree”. Each bar shows how many respondents selected each level, with the median impact score of each factor displayed below. The median is calculated by assigning numerical values to responses (1 = strongly agree; 5 = strongly disagree).

In contrast, 16 companies (45.7%) disagreed with the notion that certification is valuable when targeting individual consumers. At the same time, only 1–4 respondents (2.9–11.4%) do not see the value of certification if the end buyer is a medical professional, an institutional buyer, a government body, or an insurer.

These findings validate the author’s hypothesis that companies do not see certification so worthwhile if the end buyers are individuals and are more likely to avoid the certification pathway compared to other buyer types (*p* = 0.006 comparing individuals and medical professionals, *p* < 0.001 when comparing individuals and institutional buyers, government or insurers). At the same time, the attitude is similarly welcoming when working with medical professionals versus institutional buyers and versus government, or insurers, with no significant statistical difference between these groups in any combination (*p* > 0.999).

Compared to the 2017 survey^8^, manufacturers’ attitudes have remained the same when the buyer persona is an individual. However, a more positive trend is observed when the buyer persona is a medical professional - with the median attitude shifting from 3 (“Neutral/Don’t know”) in 2017 to 2 (“Agree”) in 2025. This suggests that medical professionals now are more knowledgeable and demanding when it comes to digital technologies. The case of institutional buyers, however, is noteworthy. Compared to 2017, a larger proportion of manufacturers now strongly agree that undergoing assessment is worthwhile - 42.9% (15 out of 35) in 2025, up from 28.6% (6 out of 21) in 2017.

### Support

**Fig. 8:**
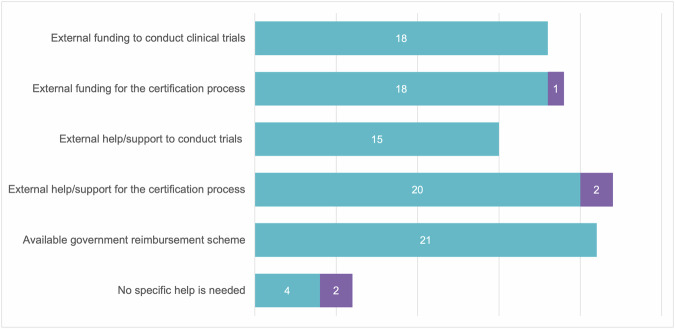
Participants’ responses on what support they would find beneficial (*n* = 35). Number of respondents for each type of support, several choices were allowed, including free form suggestion.

The final multiple-choice question asked respondents what kind of support they would find helpful in their business operations. Their answers offer direct suggestions to policymakers and other stakeholders on how to better support manufacturers and foster industry development (Fig. 8).

The most beneficial form of support reported was assistance with the certification process, which 63% of companies, both SMEs and large enterprises, would find helpful. An available government reimbursement scheme was considered useful by slightly fewer respondents, 60%, though it was particularly important for SMEs, as only they reported needing it. External funding, both for the certification process and to conduct clinical trials, was seen as helpful by at least half of the respondents (54.35% and 51.4%, respectively). Six companies (17.1%) reported that they did not require any support.

Bar chart illustrating the types of support manufacturers would find most useful. The support options are listed in descending order, with the most valued at the top. Blue represents SME respondents, while purple represents large companies. Each bar indicates how many respondents selected each type of support.

Respondents were given the opportunity to provide open-ended suggestions on the types of support they would find most helpful, and three responses were collected. One company suggested improvement for the recertification process: ‘*Initial certification with PMS/PMCF but without each 5 years recertification reviews*.’ Another respondent expressed the need for ‘*External help/support to clinical evaluation*.’ A third emphasized the need for regulatory clarity, noting: ‘*Clarity to classification of SaMD. Huge differences btw [between] different EU countries*.’

## Discussion

The research findings confirm the previously theoretically identified hindering factors for SaMD development and deployment, responding to the first Research question. The new regulation has a substantial impact by adding additional costs, creating challenges in generating clinical evidence as there are higher expectations and requirements for all of them, even for low-risk devices, causing delays in market entry, and complicating software update deployments. Companies struggle throughout the process, as there is a lack of knowledge and guidance. The findings also support the previous claim that the new regulation for many companies means upclassification from Class I to Class II or higher^14^, which brings an additional layer of burden for many companies, especially for SME.

The research findings also highlight the negative effect of the MDR on the industry, responding to the second Research question. A considerable proportion of companies have canceled new product launches, and others have discontinued an existing product. Many of the companies reported exiting the EU market in favor of other regions, and even shutting down their companies. These findings support the previous narrative that the manufacturers are withdrawing their products due to the MDR^15,16^.

This is also an alarming signal that innovation is slowing in the EU and that the availability of medical devices is declining. For the European market, this also means that the companies are moving their businesses to other regions, along with the tax revenues, jobs, and in many cases, talent. This shift can ultimately also harm the patients in the EU, who may no longer have access to the latest innovations in the medical device industry. In the long term, without any action and support, the EU could experience an overall decline in innovation, market stagnation, and lower healthcare quality than in other world regions.

Additionally, in response to the third Research question, many companies do not see value in obtaining the medical device certification when the end user is an individual consumer. This finding supports the hypothesis that it is a strategic business decision to avoid the regulatory pathway. However, such an approach may be harmful to society in the long term, as it allows many unregulated wellness products of potentially questionable quality to enter and operate in the market. A significant proportion of these wellness solutions have been developed without the involvement of health professionals, many lack rigorous data on their efficacy and effectiveness, and a substantial number remain unsupported by empirical evidence. At the same time, real-world examples indicate that certain digital interventions may cause harm, with some instances reportedly contributing to users engaging in suicidal behavior^11^. Without any regulatory oversight, this practice is likely to emerge and intensify over time. It is therefore crucial that wellness products comply with at least minimum quality standards. The author proposes supplementing the General Product Safety Regulation with specific provisions for products intended for health-related use, such as mandatory expert involvement in content development and providing users with easily accessible information supporting the product’s claims.

Acknowledging the profound impact of the new MDR on manufacturers, it is important to support companies in their efforts to develop high-quality products with health benefits. In this regard, the findings indicate that companies would welcome various forms of support, with assistance during the certification process being especially valued. This suggests that companies seek not only financial support but also knowledge-based support, which could take the form of educational or community hubs, cooperation with academic institutions, or specific accelerators, educational programs, and such. Additionally, the availability of a government reimbursement scheme, particularly important for SMEs, could help keep at least some companies on the certification pathway, rather than choosing to avoid certification when the end user is an individual consumer, as highlighted above. These government reimbursement schemes are important not only for existing companies to maintain their operations and reach a larger market segment that might otherwise be unreachable because of the price, but also for newly established companies. The potential access to the market can help these new companies attract investors during the early stages of product development.

It is also important to reconsider how we approach and perceive regulation - whether we are prepared to take proactive steps to improve the environment and support the development of innovative solutions. Stern, in her study, found that medical device pioneers spend 34% more time navigating regulatory processes than their followers - not due to their technological advancements, but because of insufficient regulatory guidance^17^. Chatterji found that the most significant advantage of new companies lies not in the previous technological knowledge, but in regulatory expertise (combined with marketing knowledge)^18^. Therefore, it is essential to find solutions that support innovators on their journey.

Recognizing both the potential and the dynamic nature of AI solutions, many countries are now creating AI sandboxes that allow developers to test their technologies in controlled environments before releasing them to the public. A notable example is the pilot program introduced by US Food and Drug Administration in 2025 for innovative digital health devices, which allows to provide care while simultaneously collecting real-world data to evaluate devices performance, yet exercising enforcement discretion for certain regulatory requirements^19^. Such an initiative promotes collaboration among stakeholders, generates valuable insights into potential risks and operational challenges, as well as allows to review and improve the regulatory oversight. Another noteworthy example is the United Kingdom’s Regulatory Innovation Office, which aims to update regulation, speed up approval times, and ensure regulatory bodies work together effectively^20^. The overarching goal is to ensure that regulation enables, rather than hinders, innovation in science and technology^21^. These kinds of initiatives help to create an agile ecosystem where the industry can rely on support at the state level. And they can be mutually beneficial, as they promote collaboration among stakeholders and ensure timely feedback to all parties, support developers in building compliance-by-design, and help governments to identify existing challenges, pain points, and regulatory gaps, meanwhile allowing to refine regulatory frameworks.

This research has several limitations. First, the EUDAMED database is not yet fully operational, so not all manufacturers are listed; therefore, not all manufacturers were reached to obtain their perspective in the survey.

Furthermore, the number of respondents was limited, and the response rate was slightly above 5%, despite two reminder emails being sent. The author decided not to send out a third reminder email because the second reminder gave only six additional responses. This low response rate may introduce non-response bias, where some of the groups are underrepresented. The relatively small sample size also imposes limits on the generalizability of the statistical findings. With the limited data, the ability to detect small or moderate effects is reduced. This means that smaller effects could have been undetected due to the limited sample size, but it does not mean their absence.

At the same time, the demographic data indicate that the proportion of SMEs among respondents aligns with that of the general population (approximately 90%). Moreover, participants represent 13 countries, providing diverse local experiences and perspectives. In addition, some of the findings are consistent with results from similar studies - for example, the proportion of companies that experienced upclassification - supporting the assumption that the respondents are reasonably representative.

Another limitation concerns the questions on the perceived value of certification depending on the type of consumer. The attractiveness of certification may vary based on the type and characteristics of the product, such as its intended use, indications, and patient characteristics. For example, manufacturers of high-risk devices may have a stronger sense of social responsibility and therefore view certification as essential, while low-risk product manufacturers would not see it worthwhile. This correlation was not studied in this research. In addition, the survey was sent out to only med-tech manufacturers, so the responses reflect their perspective alone. Including developers of wellness products could provide a different picture, as it can be hypothesized that a greater proportion of these developers would consider certification less attractive.

## Methods

### Search strategy

The author conducted an online survey, which was emailed to all SaMD manufacturers identified in the EUDAMED database^22^. Eligibility criteria included manufacturers with a medical device type classified as “Software” and operating under either the MDD or the MDR. The respondent list was compiled in February 2025, consisting of 691 unique email addresses; the survey invitations were sent out in April 2025, with two reminder emails sent in May 2025.

### Survey design

The survey was structured into three parts. The first part collected information about the respondent’s company, products, and target markets. The second part focused on the impact of the MDR, with questions formulated based on the author’s previous theoretical work. The first question included five statements evaluated on a 5-point Likert scale, ranging from “no impact” to “severe impact,” using a unipolar approach to measure a single attribute - the level of impact. The second question contained three statements assessed on a 5-point Likert scale from “strongly agree” to “strongly disagree,” followed by one multi-answer question. These three questions would help to answer the first Research question. The following two multi-answer single-choice questions and two binary questions would help to answer the second Research question. The final question in the second part assessed certification attractiveness through four statements, which were also rated on a 5-point Likert scale from “strongly agree” to “strongly disagree.” This question would help to answer the third Research question.

The third and final section of the survey included one question aimed at understanding the type of support manufacturers would find most beneficial. The answers to this question would provide guidance to policymakers on how to support manufacturers and the industry as a whole and how to prioritize actionable initiatives.

The survey was reviewed by two independent experts - a statistician and a social anthropologist - who provided feedback on wording and structure, and some questions were adjusted for better clarity. The survey was developed using the Typeform online tool and took approximately 6 minutes to complete. The survey questions are available as Supplementary material. All recipients of the email and survey were informed of the purpose of the survey and that the data would be processed solely for research purposes, in compliance with the GDPR; completion of the survey served as consent.

### Data analysis

The survey data were exported from Typeform and then analyzed using Microsoft Excel and IBM SPSS Statistics. Data were presented using absolute numbers, percentages, and graphical visualizations. Medians were reported for Likert scale items to represent tendency. Group differences were assessed using the Mann–Whitney U test, comparing (1) companies whose products were upclassified versus those that were not, and (2) respondents with versus without prior experience under the MDD. A probability of *p* < 0.05 was considered statistically significant.

## Supplementary information

Supplementary Material

## Data Availability

The datasets generated and analyzed during the current study are not publicly available due to privacy concerns but are available from the corresponding author on reasonable request and approval from the ethics committee of the Riga Stradins University.
