# Supplementary material for: Impact of the new European medical device regulation on digital health manufacturers
Source: NPJ Digit Med. 2026 May 26;9:638. doi: 10.1038/s41746-026-02812-1 (PMC13486492; doi:10.1038/s41746-026-02812-1)
Supplement: Supplementary file 1 [file 41746_2026_2812_MOESM1_ESM.pdf]

Survey questions

Part 1. Information about your company.

1. Country of your company: [dropdown country list]

2. Your main markets: [multiple choice country list]

3. Company size:

Company category	*Staff headcount*	*Turnover*	*or*	*Balance sheet total*
SME	< 250 employees	≤ € 50 million		≤ € 43 million
Large	≥ 250 employees	> € 50 million		> € 43 million

   - ☐ SME
   - ☐ Large

4. Number of currently operating products (regardless of their certification status):
   - ☐ One product
   - ☐ Two or more products

5. Regulatory experience:
   - ☐ Company has (or has had) at least one certified product under the Medical Device Directive 93/42/EEC
   - ☐ Company has (or has had) at least one certified product under the Medical Device Regulation EU 2017/745

Part 2

This part consists of questions about how the new MDR impacted your company's operations.

1. Please assess the impact of the MDR…

	No impact	Minor impact	Moderate impact	Significant impact	Severe impact
… **on the additional time required to launch a product** *(no impact = no additional time was needed // severe impact = a lot of additional time*					

was required, causing significant and long launch delays)					
… **on clinical evidence requirements** *(no impact = no problems in providing the required evidence // severe impact = we needed to conduct additional trials, utilized a large amount of resources, the process was challenging)*					
… **on additional costs needed** *(no additional costs occurred // severe impact = extremely high costs occurred, severely impacting our business)*					
… **on the classification process and class selection** *(no impact = we were certain if the product is a medical device // severe impact = we experienced conflicting information and spent a lot of resources to understand if the product is a medical device)*					
… **on software updates** *(no impact = our processes are unaffected, development is at the same velocity as it normally would // severe impact = our development velocity is severely impacted)*					

2. Please assess how much you agree with the proposed statements:

	Strongly agree	Agree	Neutral	Disagree	Strongly disagree
We had enough knowledge of regulatory requirements and processes					
We had enough regulatory guidance and clarity					

3. Please assess how much you agree with the proposed statement: "We experienced problems with a Notified body (their capacity is low, they are not accepting new clients, etc.)":
   - ☐ Strongly agree
   - ☐ Agree
   - ☐ Neutral
   - ☐ Disagree
   - ☐ Strongly disagree
   - ☐ NB involvement was not needed (having only Class I devices)

4. Was your SAMD product up-classified, compared to the MDD?
   - ☐ Yes, from Class I to Class II or higher
   - ☐ No, it stayed in the same Class
   - ☐ We did not operate under the MDD

5. Have you canceled a new product launch due to the MDR?
   - ☐ Yes
   - ☐ No
   - ☐ We had no plans to launch a new SAMD product

6. Have you reduced your product portfolio (discontinuing at least one product) due to the MDR?
   - ☐ Yes
   - ☐ No
   - ☐ We have always had only one product

7. Have you exited or are you in the process of leaving the EU market for at least one product while continuing to sell that product in other markets?
   - ☐ Yes
   - ☐ No

8. Are you planning, or at least considering, shutting down operations due to the regulatory burden?
   - ☐ Yes
   - ☐ No

9. Please rate the statement: "It is worthwhile to obtain CE certification if… “
   Please rate it even if you currently do not operate under that business model

	Strongly agree	Agree	Neutral	Disagree	Strongly disagree
the customers are **individuals**					
the customers are **medical professionals** (doctors, nurses, pharmacists)					
the customers are **institutional buyers** (hospitals, clinics, etc.)					
the customers are **government or insurers**					

Part 3

What help would you need for your business in the context of the MDR?

- ☐ External funding to conduct clinical trials
- ☐ External funding for the certification process
- ☐ External help/support to conduct trials (such as knowledge, expert placement, etc.)
- ☐ External help/support for the certification process (such as consultations from compliance experts, support for documentation, etc.)
- ☐ Available government reimbursement scheme to ensure demand for our product
- ☐ No specific help is needed
- ☐ Other:
